# Breastfeeding inequities in South Africa: Can enforcement of the WHO Code help address them? – A systematic scoping review

**DOI:** 10.1186/s12939-021-01441-2

**Published:** 2021-05-04

**Authors:** Debbie Vitalis, Mireya Vilar-Compte, Kate Nyhan, Rafael Pérez-Escamilla

**Affiliations:** 1 Yale University School of Public Health, New Haven, CT 06510 USA; 2 Yale University, Cushing/Whitney Medical Library, 333 Cedar St., New Haven, CT 06510 USA

**Keywords:** Breastfeeding, The WHO code for Marketing of Breastmilk Substitutes, HIV, Infant feeding, Infant feeding guidelines, South Africa

## Abstract

**Introduction:**

Suboptimal breastfeeding rates in South Africa have been attributed to the relatively easy access that women and families have had to infant formula, in part as a result of programs to prevent maternal-to-child transmission (MTCT) of HIV. This policy may have had an undesirable spill-over effect on HIV-negative women as well. Thus, the aims of this scoping review were to: (a) describe EBF practices in South Africa, (b) determine how EBF has been affected by the WHO HIV infant feeding policies followed since 2006, and (c) assess if the renewed interest in The Code has had any impact on breastfeeding practices in South Africa.

**Methods:**

We applied the Joanna Briggs Institute guidelines for scoping reviews and reported our work in compliance with the PRISMA Extension (PRISMA-ScR). Twelve databases and platforms were searched. We included all study designs (no language restrictions) from South Africa published between 2006 and 2020. Eligible participants were women in South Africa who delivered a healthy live newborn who was between birth and 24 months of age at the time of study, and with known infant feeding practices.

**Results:**

A total of 5431 citations were retrieved. Duplicates were removed in EndNote and by Covidence. Of the 1588 unique records processed in Covidence, 179 records met the criteria for full-text screening and 83 were included in the review. It was common for HIV-positive women who initiated breastfeeding to stop doing so prior to 6 months after birth (1–3 months). EBF rates rapidly declined after birth. School and work commitments were also reasons for discontinuation of EBF. HIV-positive women expressed fear of HIV MTCT transmission as a reason for not breastfeeding.

**Conclusion:**

The Review found that while enforcing the most recent WHO HIV infant feeding guidelines and the WHO Code may be necessary to improve breastfeeding outcomes in South Africa, they may not be sufficient because there are additional barriers that impact breastfeeding outcomes. Mixed-methods research, including in-depth interviews with key informants representing different government sectors and civil society is needed to prioritize actions and strategies to improve breastfeeding outcomes in South Africa.

**Table:** 

This article is a part of the Interventions and policy approaches to promote equity in breastfeeding collection, guest-edited by Rafael Pérez-Escamilla, PhD and Mireya Vilar-Compte, PhD	

## Introduction

In 2016, South Africa recorded 67.3% of infants initiating breastfeeding within 1 h of birth, and only 31.6% being exclusively breastfed, with a mean exclusive breastfeeding duration of 2.9 months [1], the lowest rates on the African continent [2]. South Africa, an upper-middle income country in Sub-Saharan Africa has a population of about 58 million [3, 4]. While South Africa is rich in natural resources, its social indices reflect structural vulnerabilities and inequities, such as healthcare gaps and the uneven impact of HIV/AIDS [5]. South Africa’s maternal mortality ratio is 119/100,000 live births [6], and child mortality rates have been steadily declining with infant and under-5 mortality rates of 28 and 35 per 1000 live births compared to Sub-Saharan Africa’s rates of 52 and 76 respectively [7, 8].

Breastfeeding is vital for an infant’s development and survival as it reduces morbidity and mortality from diarrhea, pneumonia and malnutrition, particularly in infants under-5 years [9–11]. Breastfeeding also reduces the risk of childhood obesity and fosters cognitive development [10, 12–14]. Furthermore, it confers health benefits to mothers including reduced risk of cancers (breast, ovarian), hypertension and diabetes [10, 15, 16].

The World Health Organization (WHO) and the United Nations Children’s Fund (UNICEF) recommend exclusive breastfeeding (EBF) of infants from birth to 6 months, followed by the introduction of nutritious and safe complementary foods with continued breastfeeding for at least 2 years [9]. The United Nations (UN) policy brief on *The UN Decade of Action on Nutrition* includes five targets to eradicate all types of malnutrition, as well as six targets to improve maternal, child health and nutrition by 2025 [17]. Target 5 on breastfeeding, aims to increase exclusive EBF rates to at least 50% by 2025 [17]. Hence, it is of concern that globally just 44% of infants are breastfed soon after birth, and 40% of those less than 6 months old are exclusively breastfed [9, 18]. In 1985, HIV infection through human milk or breastfeeding was first recognized in the United States [19, 20] and the Centers for Disease Control and Prevention issued guidelines for HIV-positive women to adopt replacement feeding [21].

In terms of HIV-positive women, WHO guidelines on infant feeding have drastically evolved over time. Initially in 2001, the WHO advised women not to breastfeed when affordable and safe human milk substitutes were available due to evidence supporting the mother to child transmission (MTCT) of HIV through human milk or breastfeeding [22]. By 2006, WHO advised that early cessation of breastfeeding before 6 months was no longer required for HIV-positive women [23]. In 2010, they advised women on anti-retroviral treatment (ART) to exclusively breastfeed for 6 months and continue breastfeeding for 1 year [10, 24]. Finally in 2016, the timeline for breastfeeding was extended to at least 24 months, and mixed feeding was no longer considered a risk factor for MTCT as long as ART was available [9].

In 2011, South Africa changed its infant feeding policy to EBF for all women regardless of HIV status [25]. This shift in policy conveyed through the Tshwane declaration was aligned with WHOs 2010 guidelines on HIV and infant feeding that realigned breastfeeding guidelines of HIV-positive women with HIV-negative women as long as ARTs are available [25, 26]. The declaration also called for not distributing free infant formula for HIV-positive women (except for medical reasons) at public health facilities, and for these facilities to become “baby-friendly” by 2015 [18, 25].

The UNICEF/WHO Baby Friendly Hospital Initiative (BFHI) launched in 1991 was designed to strengthen the capacity of maternity facilities to protect, promote, and support successful breastfeeding [27]. The Tshwane declaration also called for the government to establish legislation to enforce the WHO International Code on Marketing of Breast Milk substitutes (The Code) [25]. The Code includes specific guidelines to protect, promote and support breastfeeding by regulating the advertising and sales of breastmilk substitutes (BMS), bottles and teats [28, 29].

While The Code was approved since 1981 by the World Health Assembly [29] violations continue to be widespread globally due to weak Code monitoring and enforcement mechanisms across countries, especially in low-to-middle income countries (LMIC) [30–32]. As a result, infant formula sales have increased exponentially in LMICs, including in South Africa resulting in higher rates of morbidity and mortality [30, 31, 33, 34].

South Africa also has one of the highest rates of HIV in the world with an adult prevalence of 19.2% [4]. Breastfeeding outcomes are suboptimal in South Africa [11, 33, 35]. Some have attributed it, at least in part, to the relatively easy access that women and families have had to infant formula as a result of infant formula distribution programs designed to prevent MTCT of HIV [36]. Apparently, this policy may have had an undesirable spill-over effect on HIV-negative women as well. Hence, South African academics and advocates have called for enforcing The Code within the framework of the most recent WHO guidelines on infant feeding in the context of HIV [9, 33]. Enforcing The Code is paramount in the context of WHOs most recent guidelines on infant feeding [9] to improve EBF. Thus, the aims of this scoping review were to: (a) describe EBF practices in South Africa, (b) determine how EBF has been affected by the HIV infant feeding policies followed since 2006, and (c) assess if the renewed interest in The Code has had any impact on breastfeeding practices in South Africa.

## Methods and analyses

We applied the PRISMA Extension (PRISMA-ScR) and Joanna Briggs Institute guidelines for scoping reviews [37, 38]. The review protocol was registered in Open Science Framework (OSF) (https://osf.io/sxcfv/). A highly experienced medical librarian (KN) conducted a peer-reviewed comprehensive search of multiple databases.

### Information sources and methods

The databases searched in this project and their platforms were: MEDLINE All (Ovid), PsycINFO (Ovid), Embase (Ovid), Global Health (Ovid), Web of Science Core Collection (as licensed by Yale University, Core Collection included Science Citation Index Expanded (SCI-EXPANDED), Social Sciences Citation Index (SSCI), Arts & Humanities Citation Index (A&HCI), Conference Proceedings Citation Index- Science (CPCI-S), Conference Proceedings Citation Index- Social Science & Humanities (CPCI-SSH), Book Citation Index– Science (BKCI-S), Book Citation Index– Social Sciences & Humanities (BKCI-SSH), Emerging Sources Citation Index (ESCI) --2015-present, Current Chemical Reactions (CCR-EXPANDED), and Index Chemicus (IC), Dissertations and Theses Global (ProQuest), Africa-Wide Information (Ebsco), CENTRAL (Cochrane Library), CINAHL (Ebsco), and Africa Index Medicus (Global Index Medicus). All titles/abstracts and texts were screened in Covidence except for the South African National ETD Portal (via netd.ac.za). This portal was searched online, but because records’ export was not possible, the potentially relevant records were screened by an author (DV) online and only records requiring full text screening were added to Covidence. Each database was searched individually, using a combination of keywords and (if available) controlled vocabulary. No study registries were searched. No citation chaining was done.

Records published between 2006 and the dates of the searches in 2020 were retrieved. This date limit was used because this was the period of major policy shifts in WHOs infant feeding guidelines in the context of HIV. No limits were imposed with respect to study design and languages. Conference papers were not retrieved. Papers with animal subject indexing were excluded in some databases, but only if they did not also have human subject indexing. This search strategy was developed independently and did not use any published or validated filters. The MEDLINE search strategy was peer reviewed by an independent medical librarian using the Peer Review of Electronic Search Strategies Guideline [39].

### Search strategies

The search strategies for each database are available in full. Search terms for Ovid Medline are presented in Table 1. While the strategies used appropriate syntax, indexes, and controlled vocabulary for the various databases, each one includes queries for the setting (South Africa) and queries for the broad topic (infant feeding). The strategies do not require an explicit reference to the WHO HIV infant feeding guidelines or The Code.

**Table 1:** Search terms for Ovid MEDLINE

1	[final searches]
2	exp Infant Nutritional Physiological Phenomena/
3	Milk, Human/
4	Infant Formula/ or milk substitutes/ or infant food/
5	(infant feeding* or breastfe* or breast-fe* or bottle fe* or bottlefe* or infant formula* or breastmilk or breast milk or wean or weaned or weaning).mp.
6	((feeding or fed or food or foods) adj2 (solid or solids or formula or baby or babies or infant or infants or infancy)).mp.
7	or/2–6
8	South Africa/
9	south africa*.mp,jw.
10	(Port Elizabeth or Bloemfontein or Johannesburg or Durban or Polokwane or Mbombela or Klerksdorp or Kimberley or Cape Town).mp.
11	(Eastern Cape or Free State or Gauteng or KwaZulu-Natal or Limpopo or Mpumalanga or Northern Cape or Western Cape or North West province).mp.
12	or/8–11
13	7 and 12
14	limit 13 to yr = “2006–2020”
15	14 not (animals not humans).sh.

Ovid MEDLINE(R) ALL, searched on April 16, 2020, and updated on June 1, 2020

### Inclusion and exclusion study criteria

For studies to be included, they needed to be peer reviewed, grey literature technical reports or theses and dissertations. We considered quantitative, mixed-methods and qualitative study designs. Studies had to be conducted in South Africa.

### Types of participants

Eligible participants were women in South Africa who delivered a healthy live birth (birth to 24 months) with known infant feeding practices.

### Interventions

We focused on two policy-level interventions, namely, the World Health Organization (WHO) Updates on HIV and Infant Feeding Guidelines (2016) [9], and The International Code of Marketing of Breastmilk Substitutes (The Code) [28, 29]. The updated 2016 (most recent) infant feeding guidelines recommend lifelong antiretroviral therapy for anyone diagnosed with HIV, including those who are pregnant and breastfeeding. It also provides guidance on appropriate infant feeding practices for mothers living with HIV [9]. Women who are HIV-positive and receiving antiretrovirals are advised to breastfeed following the same breastfeeding recommendations for HIV-negative women. The objective of The Code initially endorsed in 1981 by the World Health Assembly of WHO was to ensure safe and adequate nutrition for infants by protecting and promoting breastfeeding. It specifically sought to regulate the marketing of products such as breastmilk substitutes including formula, other types of milk, beverages and equipment (bottles, teats) [28, 29].

### Outcomes

The infant feeding/breastfeeding outcomes considered were: Breastfeeding initiation within 1 h of birth; Exclusive breastfeeding up to 6 months; Any breastfeeding up to 12 months of age; and Continued breastfeeding from 12 to 23 months.

### Screening

All references retrieved were first de-duplicated by KN, then uploaded into Covidence [40]. Two independent reviewers (DV and MVC) initially screened a sample of 20 references (titles/abstracts) to ensure consistency and measure inter-rater literature screening reliability.

### Data collection and analyses

#### Data extraction

Data were independently extracted and entered on a standard Microsoft Excel spreadsheet form by two authors (DV and MVC). Studies which did not meet inclusion criteria and outcomes were excluded. Any disagreement was resolved in consultation with the senior author (RPE). Data extraction fields included author names, journal name and year of publication, study design, location of study, sample characteristics, number of participants, and outcomes. These outcomes included: Breastfeeding initiation within 1 h of birth; Exclusive breastfeeding up to 6 months; Any breastfeeding up to 12 months of age; and Continued breastfeeding from 12 to 23 months.

#### Assessment of methodological quality of the included studies

We did not evaluate the methodological quality and risk of bias of the included studies since this is not required for a scoping review [37, 38].

#### Analyses and reporting

Our findings are reported based on the PRISMA Extension and Joanna Briggs Institute guidelines for scoping reviews. We analyzed the data extracted from included studies based on the HIV status of participants and their infant feeding practices and outcomes wherever provided.

## Results

**Fig. 1:**
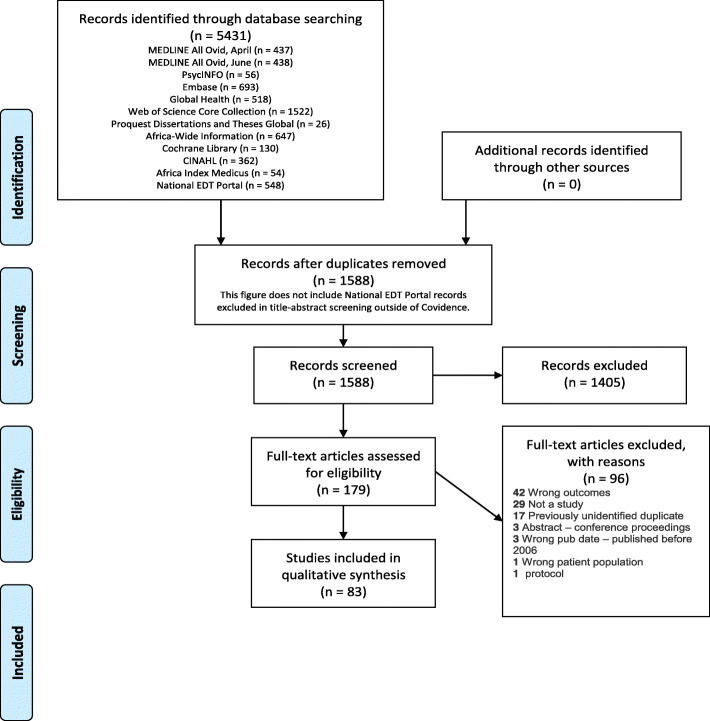
PRISMA flow diagram)

A total of 5431 citations were retrieved from eleven databases. Duplicates were removed in EndNote and by Covidence. Records from all the databases except National EDT Portal underwent title-abstract screening in Covidence; records from National EDT Portal underwent title-abstract screening on the National EDT Portal website. 35 National EDT Portal Records were identified as deserving full text screening and were added to Covidence at that stage. 1588 unique records were processed in Covidence and 179 records met the criteria for full-text screening. The final number of included records was 83 (Fig. 1 PRISMA flow diagram).

### Characteristics of included studies

Of the 83 studies included, nine were qualitative, 11 RCTs, and 63 observational. All nine South Africa provinces were represented; the highest number of studies were conducted in Kwa-Zulu Natal (34) and the lowest number in Northern Cape (2). Other studies were in Western Cape (25), Gauteng (15), Eastern Cape (14), Limpopo (13), North West (7), Mpumalanga (6) and Free State (5). Studies were conducted primarily in rural (31) and urban (25) settings; other settings included semi-rural (3), semi-urban (6) and peri-urban (22).

While the articles were published between 2006 and 2020, we interpreted findings in the context of the time period when the study was conducted. The number of articles were about evenly divided between the two periods of major changes in WHOs breastfeeding guidelines for HIV-positive women; prior to 2011 (*n* = 40 articles) and from 2011 onward (*n* = 43 articles).

### Infant feeding practices in South Africa (2006–2010)

The studies conducted during 2006–2010, i.e. before the release of the 2010 guidelines recommended HIV-positive women on ARVs to exclusively breastfeed for the first 6 months after birth (Table 2) revealed that HIV-positive women with 0–6 months infants mainly practiced formula and mixed feeding [41–44]. EBF rates in this age group ranged from 13 to 77% [44, 45].

**Table 2:** Included studies 2006–2010

Study	Participants	Infant feeding Outcomes	Findings
Qualitative			
Doherty et al., 2006	40 HIV+ women; mean age 24y; community health workers.	25 women EBF, 15 EFF. 80% (20) women who had chosen EBF introduced other liquids within the 1st month.	Women who chose to EFF had problems accessing formula milk; women were only able to maintain exclusive feeding practices for a short time due to a variety of constraints; Those who chose FF reported concern about HIV infection was the top reason for their infant-feeding choice.
Doherty et al., 2006	27 HIV+ women; mean age 25y.	2 (18%) who initiated breastfeeding were still EBF at 12 wks.; 3 stopped between 6 and 12 wks.; Formula-feeders: 88% (14) did not give breast milk to their infants.	HIV-positive women encounter many challenges in maintaining exclusive infant feeding, such as insufficient support from health staff and family pressure.
Sibeko et al., 2009	11 women (HIV+ and HIV-), mean age 25y	FF – 4 women; MF – 5; EBF – 2 women. 25% (1/4) women who chose to formula feed was able to EFF.	Non-disclosure of HIV status influenced feeding choices, which resulted in mixed feeding. Bad infant feeding advice created confusion and resulted in subpar practices such as mixed feeding.
Andreson et al., 2013	14 HIV+ women & buddy pairs, > = 18y; data for 12 women	Study end date (2 to 6 months infants), − 6 women were EBF and 6 were FF.	Buddies can provide good support for HIV+ women.
Randomized Controlled Trials			
Bork et al., 2014	751 infants (366 boys, 385 girls)	Ever breastfed: Durban −57.1% (101), KwaZulu 80.9% (72). Breastfeeding (4-6 months): 41, 53% for Durban and KwaZulu- Natal, respectively.	Not breastfeeding was associated with increased risk of serious infections particularly between 0 and 2.9 months.
Ijumba et al., 2015	1821 (intervention) and 2136 (control) pregnant women, median age 23y. 30 Com. Health workers (CHW): 15 Intervention and Control group respectively;	76% (1242/1629) women in intervention and 74% (1380/1865) women in control initiated breastfeeding after birth; 34.4% (561) intervention and 32.5% (607) control breast-fed within 1 h of birth.	CHWs had positive effect on EBF, particularly on HIV- women.
Bork et al., 2013	1028 HIV+ mother–infant pairs	56% (565) women were still breastfeeding at 3 months postpartum: 30% EBF, 18% predominant breastfeeding, and 8% partial. EBF: 90.4, 73.1, 69.9, 56.8, and 80.0% for Bobo-Dioulasso, Mombasa, Nairobi, Durban, and Somkhele, respectively.	Overall, EBF was brief, particularly for boys.
Cournil et al., 2013	795 HIV+ women	77.7% 618 initiated breastfeeding at birth; 22.3% (177) mothers never breastfed.	By 6 months post-delivery, infants weaned/not breastfed had approximately seven-fold higher risk of dying compared with infants being breastfed.
Tylleskär et al., 2011	2579 mother-infant pairs	EBF prevalence in South Africa at 12 weeks (24-h recall) in the intervention and control groups: 10% (56/535) 6% (30/485), respectively.	No differences found for the prevalence of diarrhea at age 12 weeks or 24 weeks between the clusters within the countries; No significant change in EBF rates for South Africa which were low at baseline.
Doherty et al., 2014	964 HIV- mother–infant pairs; mean age 24y in Rietvlei and Umlazi and 25y in Paarl	34% (114/330), 20% (57/283) and 36% (127/351) of HIV-negative women in Paarl, Rietvlei and Umlazi, respectively, stopped breastfeeding before 6 months postpartum.	For HIV-unexposed infants: low birthweight and short rates of breastfeeding increased risk of hospitalization/death during 1st 6 months of birth.
Doherty et al., 2012	Sub-group analysis of data for 999 women who ever breastfed; median age 22y in Rietvlei and Umlazi; 24y in Paarl.	At 12 weeks postpartum - 20% HIV- and 40% HIV+ women had stopped breastfeeding; 97% HIV- women and 42% HIV+ women ever breastfed.	Less than optimal early feeding practices; Early cessation of breastfeeding occurs among both HIV- and HIV+ women.
Engebretsen et al., 2014	2579 mother-infant pairs in 3 countries: 794 - Burkina Faso, 765 – Uganda, 1020 - South Africa	< 4% women in Burkina Faso and about 50% in South Africa initiated breastfeeding within the 1st hour of birth.	Behavior change may not have occurred in South Africa.
Ramokolo et al., 2015	641 HIV unexposed children, median age 22 months	Infants using any breast milk changed from 89.3% (3 weeks) to 79.4% (12 weeks); Cereal use at 12 weeks −79.5% who were not breastfed, and 59.6% who were breastfed.	Infant feeding actions in the first 12 weeks can predict the development of childhood overweight and obesity.
Observational			
Goga et al., 2009	665 HIV+ & 218 HIV- women (age range 21–30) and infants;	Complete breastfeeding cessation (CBC) HIV+: 43.6% (88) reported CBC by 24 weeks.; HIV-neg: 97% initiated EBF.	Although national guidelines advise HIV+ women to breastfeed, they stop by 24 weeks; 39–44% of women practicing EBF/PBF at week 3 complied with recommendations.
Becquet et al., 2009	2190 HIV+ women, aged ≥16 years	90% of infants in both studies continued to be breastfed by 3 months of age; Mixed feed - 22% of infants by 3 months of age.	Breastfeeding duration is the primary contributor to HIV postnatal transmission; risk is quite similar for both exclusive & predominantly fed infants; Risk of postnatal HIV infection was 3.9% for infants breastfed less than 6 months, and 8.7% for those breastfed for at least 6 months.
Bland et al., 2007	2491 women (1253 HIV+, 1238 HIV-); median age 25	HIV+ women (EBF −78%; replacement feeding 42%; HIV- women: 75% maintained intentions to EBF and < 1% (11) infants were not breastfed.	For most women counselling helped with matching infant feeding intentions based on existing resources for appropriate feeding. Most HIV+ women did not have resources for safe replacement feeding, so they decided to EBF.
Bland et al., 2008	HIV-women (1219 infants); HIV+ women (1217 infants);	Median duration of EBF: HIV-, 177 days; HIV+, 175 days; EBF at 3 & 5 months: HIV- women (83.1, 76.5%); HIV+ women (72.5, 66.7%) respectively.	Both HIV+ and HIV- women can maintain EBF for 6 months with support in the home from trained lay counselors.
Chetty et al., 2014	2340 women (1197 HIV-, 1143 HIV+); Median age: HIV- (21.8y); HIV+ (25.1y)	Median duration of EBF: HIV- (179 days); HIV+ (175 days); Birth to 5 months feeding patterns: EBF – HIV- 76.9% (920); HIV+ 66.7% (762); Mixed feeding - HIV- 11.9% (143); HIV+ 8.8% (101); No breastfeeding – HIV-10.4% (125); HIV+ 24.1% (275).	Breastfeeding did not increase postpartum weight loss; HIV+ women lost less weight during 1st 6 months & 12 months postpartum than HIV- women.
Coovadia et al., 2007	2722 HIV+ and HIV- pregnant women, median age, 25.1y	83% (1132/1372) HIV-exposed infants initiated EBF from birth; median time for cumulative EBF, 159 days. EBF at 26 weeks – 37% (415/1132).	Mixed breastfeeding increased HIV transmission risk; EBF infants were less likely to get HIV than breastfed infants who used solid foods; HIV+ women can receive support to EBF.
Doherty et al., 2007	635 HIV+ mother–infant pairs, mean age: Intent to EFF, 25.8y; Intent to EBF, 25.3y	13% who intended to breastfeed were EBF at 12 weeks; 42% mixed feeding; Predominantly breastfeeding (11%); EFF (33.5%).	Risk of getting HIV or death in both breastfeeding groups was high due to low rates of EBF; Inappropriate infant feeding choices were made based on the availability of 3 factors (piped water, electricity, gas or paraffin for cooking fuel, and early disclosure of HIV status).
Ghuman et al., 2009	168 HIV+ & HIV-women, mean age 24y	97% (163) infants got breast milk as their first feed, 3% (5) were formula-fed; At 14 weeks: EBF was 18%; 52% got water; and 73% solids; 87% (20/23); Week 14: 11% HIV+ women were EBF, and 63% (12/19) mixed feeding.	Most women were not adhering to recommended infant feeding guidelines by 14 weeks of age; Women were unable to maintain EBF. HIV+ mothers breastfed at birth and more likely to formula feed than HIV- women.
Goga et al., 2012	665 HIV+ and 218 HIV- women; Median age: HIV-pos 25; HIV-neg 23	EBF at 3 weeks: HIV+ 42% (130) vs HIV- 17% (33); 47% (271) HIV+ women reported no breastfeeding; HIV- women at weeks 3 and 12: 17% (33) and 3% (5) practiced EBF.	While feeding practices were subpar among both groups of women, HIV-positive women engaged in more safer practices.
Matji et al., 2009	222 HIV+ and 53 HIV- women;	At 6 weeks: 94% HIV- mothers were breastfeeding, 69% HIV+ mothers were FF; Intro of food by 6 weeks: 14% HIV- mothers; 1 HIV+ mother had stopped breastfeeding by 6 weeks.	Influences in the home environment resulted in changes in infant feeding practices.
Patil et al., 2015	2053 infants; median age 3–12 days	Breastfeeding initiation within 1 h - 59.7%; EBF at 30 days - 29.5%; Partially Breastfed at 30 days - 36.6%; Completely weaned by 30 days - 2.6%.	Shift from EBF in the first month of life. Liquids and solids were usually given to infants in the first month.
Rollins et al., 2013	2789 women; Median age: HIV-pos 25.0y; HIV-neg 21.7y	81.4% HIV+ and 92.9% HIV- mothers EBF at 6–8 weeks; 61.8 and 72.6% at 3–4 months; median time for cessation of breastfeeding-171 days.	EBF was associated with less adverse events with mixed feeding or not breastfeeding in both HIV exposed and unexposed infants.
Rossouw et al., 2016	47 mother–infant pairs, 25 HIV-exposed and 22 HIV-unexposed infants	HIV-exposed infants: One mother initiated breastfeeding and continued up to 18 months; HIV-unexposed infants: all mothers initiated breastfeeding with > 50% within an hour of birth. 62 and 52% of HIV-unexposed mothers were breastfeeding at 12 and 18 months respectively. No infant EBF at 6 months.	Among both groups, there was low compliance with breastfeeding guidelines and dietary diversity. Breastfeeding rates were low in HIV-exposed infants due to free formula distribution at health facilities.
Ahmadu-Ali et al., 2013	386 women, mean age 25y	At 6 weeks: 53.1% (205) women were EBF; 26.6% (103) EFF; 20.3% (78) mixed feeding. EBF at 6 weeks: 52.7% (157) – HIV-neg vs 60.6% (43) HIV-pos.	HIV- women reported more counselling during antenatal care than HIV+ women; EBF was lower in HIV+ than HIV- women.
Faber et al., 2007	505 infants, mean age 9 months; 441 mothers, 64 caregivers, mean age 25y	Breastfeeding initiated - 96% infants; breast milk only - 58%; mixed feeds - 23%; bottle feeds only - 18%. 61% infants had solid foods before 6 months.	Exclusive breastfeeding to age 6 months was rarely practiced. Trained community health workers should help with poor infant feeding practices and micronutrient deficits.
Kyei et al., 2014	2660 women, 13–50 years	42.5% vs 57.5% women were still breastfeeding; 70.6% of those who stopped breastfeeding breastfed < 24 months.	Study showed duration of breastfeeding in Vhembe district decreased from > 24 months to just 18 months.
Ladzani et al., 2011	815 HIV-positive women; mean age - 27.7 years	50% EFF, 35.6% EBF, 12.4% mixed feeding. EBF within 1 h of delivery - < 50%; EFF within 1 h of delivery - > 50%.	Knowledge gaps of PMTCT and infant feeding policy contributed to inappropriate feeding choices. Variables associated with mixed feeding: Vaginal delivery; infant hospitalization, and currently pregnant. Variables associated with FF: older age, knowing the HIV status of the infant; and higher HIV transmission/breastfeeding knowledge.
Ukpe et al., 2009	33 mothers & infants; Mean age women − 30.7y; Mean age infants - 3.5 months.	ERF - 50% (15/30); EBF - 27% (8/30); − Mixed feeding 23% (7/30).	ERF was the most frequent infant feeding practice. Women who FF used different types of commercial milk. Quality of counselling should be strengthened to enhance infant feeding practices.
Yako and Nzama, 2013	60 women, mean age 26.5y; 53% (32) were HIV-negative and 46.7% (28) were HIV-positive	At six weeks: EBF group – 13.3% (8/19) breastmilk only; mixed feeding – 11.7% (7/19). EFF group – formula only 30% (18/41); Mixed feeding – 15% (9/41).	Educate mothers on best infant feeding practices, including non-introduction of foods/liquids at inappropriate ages.
Zunza et al., 2011	95 HIV-positive mother-infant pairs, Mean age, 27y	EFF - 97% (62); Formula – 78% (50); EBF – 2 women; Mixed feeding – 19% (12).	Advice needed on breast health during breastfeeding period and optimal infant feeding practices.
Gbadamosi et al., 2017	186 infants, ages 1 to 12 months	0.6% infants were EBF for > 3 months; 78% breastfeeding at 9 months; 39.5% mixed feeding by end of 1st month; 0.6% EBF > 6 months.	Complimentary foods provided at an early age; EBF was rarely practiced; Interventions needed to support and promote recommended infant feeding guidelines.
Aku A., 2013	125 HIV-positive mother-infant pairs, mean age, 27.8 years	Replacement feeds −84.3%; Mixed feeding −11.2%; Intro of solids − 10.4%; Mean age for intro of solids - 47 days.	Infant feeding choices influenced by family; SES factors affected growth and nutrition of HIV-exposed infants.
Abusomwan, Osaigbovo Ebenezer, 2011	395 HIV-pos mothers; 14 to 49 years	EBF - 77.7%, 6 weeks after delivery; Mixed feeding - 3%; replacement feeding - 19.3%.	EBF was the primary infant feeding choice and practice; Hardly any mixed feeding occurred in this group of HIV-positive women.
Jacobs-Jokhan, D., 2011	200 HIV-pos women, mean age 30y	EFF - 84.5%; EBF −14%; Mixed feed − 1.5%.	Study showed that babies born to mothers who did not receive infant feeding counselling were twice as likely to be HIV positive; Infant feeding counselling is necessary component of antenatal care; HIV-pos women should be counselled soon after diagnosis and throughout care.
Masters, D., 2006	42 HIV-pos women, < 19 to 49 years	Exclusively formula milk and water - 52% (22); Mixed feeds - 48% (40).	Cultural norms influence infant feeding practices, particularly introduction of solids/liquids. Women EFF wanted to prevent HIV transmission.
Mushaphi et al., 2008	185 mother-infant pairs; mean age, 25.83y	EBF (0–6 months) - 7.6% of women; 97% - still breastfeeding; 3% had stopped; 43,2% gave solid foods at three months, and 15% < 2 months.	Early introduction of other foods; Although breastfeeding was practiced by many of the mothers, EBF was rare.
Some et al., 2017	1225 mother-infant pairs- all sites; 222 from East London, South Africa; > 18 years	EBF- first 3 days - 93.4% (199); Mixed feeding first 3 days - 2.3% (5). Breastfeeding initiation within 1 h of birth – 57.7%. Median duration of any breastfeeding was 40.6 weeks.	More mothers in South Africa had to return to work after a few months, stopping them from continuing to breastfeed; Improvements needed in breastfeeding and complementary feeding of children, particularly those who are HIV-exposed.

Legend: *EBF* Exclusive breastfeeding, *EFF* Exclusive formula feeding, *MF* Mixed feeding

During this period, it was common for HIV-positive women who initiated breastfeeding to stop doing so prior to 6 months after birth (1–3 months) [46–49]. Low rates of breastfeeding were observed for a variety of reasons including free formula distribution, not wanting to transmit the virus to the infant, and work/school obligations [43, 50–53]. However, for HIV-positive women who practiced exclusive formula feeding (EFF) initially, lack of access to infant formula was one of the reasons for mixed feeding [54, 55].

While breastfeeding initiation rates were high among HIV-negative women, ranging from 52.7 to 97% [48, 49, 56–59], EBF rates declined as liquids and solids were introduced before the infants were 6 months old [60–63]. Other reasons for infant formula use included non-disclosure of HIV status, family pressure, and cultural practices [41, 54, 55, 57].

### Infant feeding practices in South Africa (2011–2020)

The post-2010 period (Table 3) after South Africa endorsed the 2010 WHO infant feeding guidelines for HIV-positive women recommending EBF for 6 months irrespective of HIV status resulted in a wide range of EBF rates among HIV-positive (26–99%) and HIV-negative (12–92%) women [64–69]. However, EBF duration was brief (1-3 months) [66, 70, 71]. While initiation of breastfeeding was high, there were low rates of EBF among HIV-negative women with introduction of liquids and other foods prior to 3 months [72–75]. In some instances, weaning occurred as early as 2 months [76]. HIV-positive women expressed fear of HIV transmission to their infants, as well as school and work commitments as reasons for discontinuation of EBF [68, 77–80].

**Table 3:** Included studies 2011–2020

Study	Participants	Infant feeding Outcomes	Findings
Qualitative			
Horwood et al., 2019	11 HIV+ women, 15-41y	EBF for 6 months – 3 women; 4 stopped breastfeeding, and 2 were MF by 6 months	Health workers influenced feeding decisions
Chaponda et al., 2017	30 HIV+ women, >18y	EBF - 50% mothers; Initiation of complementary foods from 1 to 4 months	Nurses primarily influenced feeding choices, followed by mothers and other relatives
Jama, Ngcwalisa et al., 2017	22 women (mixed HIV status); median age 25.5y.	EBF for the 1st 6 months – 23% (5/22); Food/fluids before 6 months – 77% (17/22)	Health workers were a strong influence on choice; All women experienced challenges including incorrect info from health staff, family pressure and having to return to school/work.
Mushaphi et al., 2017	37 caregivers, ≥ 16y;	Over 90% initiated breastfeeding after delivery; EBF by 3 months - < 1% No infant was EBF for up to 6 months; Liquids/solids by the 2nd month- 100%	EBF up to 6 months was not practiced; most women believed that breast milk alone was inadequate for baby’s needs and introduced water and other foods before 6 months.
Ntuli and Modibedi, 201	32 HIV-positive women, 22–38 years	EBF - 68.8% women; EFF - 31.3%; MF −5 mothers	Healthcare staff, SES, past participation in PMTCT and fear of infecting the baby, impacted mothers on infant feeding choices; Counselling needed throughout the perinatal period to enable mothers to choose and maintain appropriate infant feeding choice
Randomized Controlled Trials			
Jones et al., 2018	1368 HIV+ pregnant women and male partners; Women’s mean age – 28y	Overall EBF – 74% women; EFF – 13%; Infant feeding at 6 weeks: EBF - 73.7%; EFF - 12.6%.	Study intervention was not effective on EBF. Male involvement, HIV disclosure, or stigma did not influence EBF
Reimers et al., 2018	619 HIV+ mothers (277 control and 273 intervention); Median age (years): control 28.8y (27.5–30.0); intervention 28.4y (27.5–29.2)	EBF rates at 22 weeks: 44.68% (105/235) control and 42.75% (109/255 intervention group.	EBF rates at the final interview (about 22 weeks postpartum) were similar for the control and intervention groups.
Observational			
West et al., 2019	Quant - 8116 HIV+ and HIV- women; Qual- 12 healthcare providers, 22 HIV+ women, median age 31y.	Initiated breastfeeding - 86.3%. 93.1% HIV+ women initiated breastfeeding vs 66.3% HIV- women. EBF: 86.3% (day 7), 73.1% (week 14) and 51.7% (month six).	Overall exclusive breastfeeding and breastfeeding intent was higher among HIV-neg than HIV-pos women. Infant feeding choices were guided by a variety of reasons including healthcare staff, finances and social pressure
Goosen et al., 2014	Cross-sectional - 140 women, mean age 26.4y; Focus groups - 65	Initiated breastfeeding −77% women; EBF - 6 94% suboptimal breastfeeding: 36% predominant breastfeeding, 27%; partial breastfeeding, 31% not breastfeeding; 44% introduced food/formula milk to infant; Intro of foods - 75% < 3 months old.	Women engaged in suboptimal infant feeding methods; EBF was hardly done and other liquids and foods introduced at an early age
Chakona,G., 2020	Survey and FGD; 84 caregiver-infant pairs (0-24 m); mean age 34.7y	1st 6 months: EBF 36%; FF - 49%	Although women knew the benefits of breastfeeding, it was hardly practiced due to cultural and other reasons; Most infants were weaned by 2 months
Zulliger et al., 2013	207 pregnant and 203 post-partum women; mean age pregnant women 28.6y and 30.0y postpartum	Ever breastfed - 22% (45) women; Ever used formula – 93% (188); Mean time cessation – 10 weeks.	Advice from healthcare staff was a strong indicator of breastfeeding; Women with running water in home more likely to breastfeed
Budree et al., 2017	1071 women, median age 25.8y;	Initiation of BF at birth – 86%; EBF for 6 months – 13%; Solid foods before 4 months – 19%; 46% HIV-exposed infants vs 96% unexposed were breastfed at birth. Women who initiated breastfeeding −26% HIV-pos vs 12% HIV-neg women did EBF for ≥6 months.	Poor dietary practices and use of non-nutritious foods were noted; EBF decreased after 3 months, early use of other foods, and low use of iron rich foods between 6 and 9 months.
Ellis, K., 2013	260 HIV+ and 251 HIV- women	By 3 months: EBF −30.9% HIV-pos; MF - 86.2% HIV-neg women.	Although early infant feeding practices were low among HIV+ and HIV- women, HIV+ women continued safe infant feeding practices for 3 months.
LeRoux et al., 2020	869 mother–infant pairs; Age: HIV+ 28y, HIV- 27y	Early initiation of breastfeeding: All - 90%; HIV-pos 87%; HIV-neg 94%; Duration of EBF (months): All 1·4, HIV-pos 1·5; HIV-neg 1·4.	Suboptimal breastfeeding practices increased risk of infections for infants.
LeRoux et al., 2018	521 mother-infant pairs, Median age: All 28y, HIV-pos women 29y, HIV-neg 28y	Median duration of breastfeeding - 6 months in HIV-exposed vs 10 months for HIV unexposed infants	HIV exposed infants might be at increased risk of cognitive and motor delays, despite being breastfed and mother receiving ART.
Zunza et al., 2018	316 mothers-infants: 188 HIV+ mothers and 128 HIV- mothers; HIV-pos b/feed 27.99y; HIV-neg formula 28.09y	All HIV-neg mothers breastfed; HIV-pos (2 weeks): Breastmilk 42%; Formula 58%.	Analysis indicates inadequate duration of breastfeeding among HIV-pos and HIV-neg mothers.
Kennedy, Y et al., 2016	132 mothers, 18-42y, with mean age 27y	At 6 weeks: EBF - 69.36%; FF - 15.21%; MF- 15.21%; Complimentary foods by 6 weeks – 15.32%.	Although women had high knowledge on benefits of breastfeeding, they made unsuitable choices
Nguyen, K., 2017	471 mother-infant pairs; Median age - 28 years	Ever EBF – 91%; Median duration of EBF- 1.5 months; EBF ≥ 4 months – 24%.	Sub-optimal levels of EBF identified; Need for breastfeeding support
Van De Venter, C., 2019	584 women; median age 28 years	Infant feeding intentions: EBF - 81%; EFF- 16%; MF - 3%. 1 week postpartum: EBF −9%; EFF, 2%; MF - 90%.	Majority of women engaged in mixed feeding; non-disclosure of HIV status impacted infant feeding practices
Tchakoute et al., 2018	749 HIV-exposed uninfected and HIV-unexposed uninfected infants	EBF at birth - 99% HIV-exposed infants vs. 92% HIV-unexposed infants.	Infants EBF had lower cumulative infectious disease incidence than those who were not breastfed; no significant difference in mortality among HIV-exposed infants and HIV-unexposed infants during the first year of life in this cohort; EBF for just 4 months had protective effects on morbidity up to 1 year.
Horwood et al., 2018	4172 caregivers (mothers, fathers and other relatives); Age > 15y	Mothers: EBF - 49.8% MF- 23.1%; No BF - 27.0%; Other caregivers: EBF −11.8%; MF- 23.4% and no breast milk - 62.3%.	Although breastfeeding practices in the study were higher than previous studies, problems persist. Breastfeeding support needed for HIV+ women and those returning to work/school.
Jackson et al., 2019	Caregiver-infant pairs - 10,182, 10,106, & 9120 in 2010, 2011–12, & 2012–2013	National EBF rates (4–8 weeks of age) were: 22.9% in 2010; 35.7% in 2011–12 and 59.1% in 2012–13.	There was an increase in early EBF among infants 4 to 8 weeks due to major national policy change in breastfeeding from 2010 to 2013. Lower odds of EBF for mothers: with high SES; HIV- positive, unplanned pregnancy, primipara, caesarean delivery, and no breastfeeding counselling.
du Plessis et al., 2016	443 mother-infant pairs; mean age 29.5y; children 9.85 months	Breastfeeding initiation - 75.2%; Infants < 6 months: EBF - 38.5%; EFF - 19.7%; BF at 12–15 months old: 32.5%	Results indicated subpar infant feeding practices with both under and over nutrition observed.
Faber et al., 2016	Children 6–24 months: 158 urban and 158 rural area	Ever breastfed: rural - 79.1%; urban - 78.5%; BF 18–24 months – 14.4%.	Dietary diversity (minimum) attained by < 25% children; High levels of animal protein & cholesterol vs low levels of fiber & plant protein for urban vs rural children (18–24 months)
Fuls et al., 2020	200 infants 6–12 months, mean age 8.54 months	Overall BF - 87%. Complementary feeding (6–8 months)- 82%; Mean BF – 7.5 months; BF > 6 months – 53.7%	6.5% children experienced feeding problems, such as oral motor dysfunction; Care giver education and health-care professional training needed on transitional feeding.
Madiba et al., 2015	202 post-natal women, mean age was 31.4y.	55.6% EBF and EFF mothers practiced MF. EFF - 56.8%.	EBF and EFF mothers had problems adhering to initial infant feeding choice. Interventions needed to address cultural practices and other factors impacting EBF among HIV-pos women
Matsungo et al., 2017	750 infants, age 6 months	EBF (≤ 6 months age) – 5.9%; EBF (6 months) – 70.1%. Intro of liquids and semi-solids was 2·5 months and 3·8 months, respectively.	Interventions to encourage appropriate infant feeding practices, needed to prevent stunting.
Motadi et al., 2019	360 participants; mean age adults - 29.3y; mean age infants - infants were 12.2 months.	Initiation of BF within 1 h of birth – 67.2%. Of the 17% of women who stopped BF: 16 and 14 stopped within 1 month and 3 months of delivery respectively.	While the women were quite knowledgeable about breastfeeding, this did not translate into appropriate practices.
Nieuwoudt et al., 2018	298 HIV-pos and HIV-neg women. Median age 29y	Breastfeeding initiation – 99.5%. HIV-pos women (infants < 3 months): EBF – 44%; FF – 28.9%. HIV-pos women (infants 3–6 months): EBF – 31.8%.	HIV-positive mothers engaged in longer EBF and FF than the HIV-neg women. Mixed feeding occurred frequently
Pillay et al., 2018	73 teenage mothers, ≤ 19 (15–19) years	Initiation of BF – 100%. BF (Visit 1) – 68.5%. 14-week visit: EBF - 50.7%; MF/No breastmilk - 49.3%.	Early cessation of breastfeeding linked to maternal age ≤ 17y; Interventions needed to promote and support EBF
Remmert et al., 2020	156 HIV-pos women, mean age 28.1y	No initiation of BF: > 50%. EBF- 28.2%; EFF- 71.4%.	Low rates of EBF; Social support and services needed to promote EBF. Primary reason for not breastfeeding was fear of HIV transmission to the child.
Seonandan & McKerrow, 2016	11 dieticians, 14 nurses & 94 caregivers (41 infants < 6 months; 26 infants 6 to 24 months, and 27 children 2 to 5 years)	Ever EBF −76%. EBF (> 3 months) – 36%; EBF (< 6 months) – 84%	Although there have been better breastfeeding rates since 2003, EBF occurs for a short time. Appropriate feeding of infants and young children varies at state hospitals. Regular training needed for staff.
Siziba et al., 2015	580 mothers/caregivers; Mean age of infants, 2.9 months.	Initiated BF within 1st hour – 90%. EBF for (infants ≤6 months) – 12%; No BF (≤ 1 month) – 40%; Mean duration of EBF – 2 months.	Interventions needed to address knowledge and increase EBF, particularly at the community levels. Work, school, health status and inadequate milk affected infant feeding practices.
VanDerMerwe et al., 2015	435 mother-infant pairs; Mean age 26y; Infants 1 day – 5 months	Emalahleni vs Mbombela health subdistricts. Early initiation of breastfeeding 57% vs. 43%; EBF 60% vs 48%; ERF - 18% vs. 33%. MF - 19% vs. 15%; Mean age for complementary foods - 50 vs 35 days.	Baby-Friendly Hospital Initiative (BFHI) in community contributed to better infant feeding practices
Frans, R., 2014	175 HIV-pos & HIV-neg mothers, 12 to 49 years	EBF - 42.3%; MF - 48.6%; Formula - 9.1%.	Issues that impacted EBF were work, school, family pressure, and knowledge deficits. Recommendation to increase individual counselling sessions.
Makwela, M., 2019	146 mothers	Initiated breastfeeding – 94%; EBF - 39%; MF - 61%; 5% stopped breastfeeding < 1 month after initiation.	While there are high rates of breastfeeding initiation, problems exist with the practice of EBF
Mandiwana, T., 2017	160 mothers, 15–40 years	EBF for 6 months – 6.25%; EBF (4–6 months) 1.5%.	Adherence to 6 months of EBF was inadequate. Complimentary foods introduced at an early age; Reasons for non EBF: not enough milk, crying baby, school or work.
Mohlajoa, K., 2016	75 HIV-positive women, 18 to 45y	Initiation of BF (immediately after birth) -67.6%. EBF for 6 months – 40%.	Interventions needed to educate women on infant feeding; Lack of support, fear of stigma and cultural norms contributed to poor EBF
Morgan and Jeggels, 2015	100 HIV-positive mothers, mean age - 29 years	EBF - 54%; EFF - 46%	Poor EBF practices; Women not aware of Government’s policy to eliminate free formula; Consistent messaging needed on infant feeding
Muravha, N., 2014	122 health staff at 40 health facilities	4 violations by 4 health workers in 7.5% (3/40) facilities (violation of Article 7.3) - receipt of free gifts.	All health workers were familiar with the International Code of Marketing of Breast-milk Substitutes. Ongoing training needed on The Code
Radebe, P., 2014	4 TV channels, 9 radio stations, 116 magazines and 10 newspapers	30 violations from 117 baby product advertisements published in 8 of 169 magazines; No violations were found from advertisements on TV, radio or newspapers	Code violations identified in (4.7%) of magazines targeting mainly pregnant women. Data needed to determine full extent of violations in the media
Siziba, L., 2014	580 mothers/caregivers with infants < 6 months	Initiation of BF within 1 h of birth – 90%. EBF – 12%; No BF – 16%	Low rates of EBF, and early intro to other foods major concerns
Other			
Author	Type of document	Issue	Findings
Lake et al., 2019	Expert Commentary	Breastfeeding in SA and the BMS Industry	Effective leadership urgently required to stem violations of BMS industry

Legend: *EBF* Exclusive breastfeeding, *EFF* Exclusive formula feeding, *MF* Mixed feeding

### RCTs testing interventions to improve breastfeeding practices and outcomes

Two major multi-country RCTs conducted during 2005–2008 among HIV-positive women and infants ≤6 months old were the Kesho Bora trial [81, 82] and the PROMISE-EBF trial [83].

In the Kesho Bora trial [81, 82], conducted in Burkina Faso, Kenya and South Africa (Durban, Somkhele), HIV-positive pregnant women were randomized into two groups who received either triple ART during pregnancy through the breastfeeding period (intervention), or short course prophylactic therapy until delivery (control). All HIV-exposed infants received single-dose nevirapine at birth. Women were also counselled to either breastfeed with cessation by 6 months or formula feed from birth (free infant formula was provided for 6 months). The main objectives were to determine rates of HIV transmission, infant survival at 6 weeks and 12 months and adverse events. Bork and colleagues [81], found breastfeeding initiation to be lower in Durban (57.1%) than rural Somkhele (80.9%) among 751 HIV-exposed infants. Overall, they found that non-breastfed infants (0–6 months) had higher morbidity risks than those breastfed, with increased risk for serious infections (e.g. severe diarrhea) between 0 and 2.9 months [81].

The PROMISE-EBF trial was a behavior change intervention to promote EBF using peer counsellors in Burkina Faso, South Africa (Paarl, Rietvlei and Umlazi), and Uganda [47, 83–86]. This counselling intervention (*N* = 2579 mother-infant pairs) included one antenatal breastfeeding peer counselling visit and four postnatal peer visits. The two main outcomes of interest were prevalence of EBF and diarrhea at ages 12 and 24 weeks. Overall, Tylleskar and colleagues [83] found that EBF prevalence (all countries) in the intervention groups at 12 weeks was double that of the control groups. However, South Africa had exceedingly low EBF rates at baseline (10%), compared with 79% in Burkina Faso and 82% in Uganda [83]. There were no significant differences in the prevalence of diarrhea (all countries) between the two groups at either 12 or 24 weeks of age [83]. Finally, the authors [83] found that while the peer counselling intervention was effective in increasing EBF rates in Uganda and Burkina Faso, it was not effective in improving breastfeeding rates in South Africa.

### Violations of the international code of marketing of breastmilk substitutes

South Africa took another bold step in 2012 to enforce The Code via legislative action (i.e., regulation R991 of 2012), which sought to regulate the sales, advertising, marketing, information and education of foods promoted for infants and young children [87, 88]. The specific objective of regulation R991 was to protect and support breastfeeding by creating an environment free from the relentless marketing strategies of BMS manufacturers, and prevent conflicts of interest among the healthcare staff or other child care providers [87, 88]. Unfortunately, this legislation has had limited positive effects as violations of the Code are still prevalent, highlighted in the three articles discussed below.

Of the three included studies which focused on Code violations, two were Master of Public Health theses [89, 90] and the other was an expert commentary from academics [33] (Table 3). Muravha [89] investigated Code violations among health workers in four Provinces and 40 health facilities and found that four health workers accepted free gifts (pens, calendars, posters) from a BMS company, despite being aware of the R991 regulations. Health workers also received education materials (leaflets, booklets) and equipment (South African water bags for adult usage) which were branded with the manufacturer’s name, but not the name of a specific product marketed by the BMS company. Radebe [90] examined media (radio, television, print) infractions and identified 30 marketing violations from 117 baby products (formula, bottles and teats) advertised in magazines targeting primarily pregnant women or families. The author reported that these numbers are likely to be underestimated, since the analysis did not include all media sources.

Finally, Lake and colleagues’ commentary [33] documented anecdotally the marketing strategies of the BMS industry and their reach to health workers and other stakeholders through sponsorships of conferences and other scientific meetings, misleading information on infant formula on company websites, health promotion materials and support for staff salaries. The authors called for improved leadership efforts in enforcing the Code and strengthening breastfeeding interventions.

## Discussion

During the early years of the HIV pandemic, recommendations for infant feeding were guided by scientific evidence indicating that when safe feeding with breast milk substitutes was universally available, as was the case in the United States and other high income countries, it was appropriate to recommend for HIV-positive mothers to not breastfeed at all [21].

By contrast, as part of their infant feeding decisions HIV-positive mothers in low income countries needed to consider the risk of their children dying if they were not breastfed as a result of having access to safe replacement feeding alternatives. Since then, HIV infant feeding guidelines have evolved over time. WHO guidelines in this area switched from exclusive breastfeeding with abrupt weaning from the breast before 6 months to current guidelines recommending EBF for 6 months followed by breastfeeding continuation for at least 12 months if the mothers have ART access [9, 22–24].

The initial guidelines implemented in South Africa in 2008 [36] provided HIV-positive mothers with widespread access to cost-free infant formula at public health facilities. Unfortunately, these polices, albeit well-intentioned may have inadvertently negatively impacted the EBF behaviors among HIV-negative mothers and led to subsequent increases in infant morbidity and mortality in South Africa and similar settings [91]. Our review indeed suggests as reported in other studies that there may have been a spill-over from an infant feeding policy driven by HIV-positive women to HIV-negative mothers explaining why EBF rates are still low in South Africa [44, 92]. Unfortunately, there is no evidence that EBF rates significantly increased after the Tshwane declaration.

One of the challenges highlighted in this review is that HIV usually coexists with poverty creating a syndemic-like effect in the lives of women, as breastfeeding was disincentivized in the early WHO guidelines, generating inequities in access to infant feeding choices. In addition, as the South African HIV infant feeding guidelines evolved and pushed for phasing out free formula distribution [25, 36], some key challenges remain: (i) changing breastfeeding behaviors, social norms and medical practices have been slower than desired; and (ii) marketing of BMS and Code violations have persisted. These factors have disproportionately affected HIV-positive mothers mainly located in rural and peri-urban areas of South Africa where not breastfeeding was associated with increased risk for serious infections (chronic diarrhea, lower respiratory tract infections) and death [45, 81, 84]. Further research is needed to address how poverty affects women’s ability to successfully implement the national infant feeding guidelines for HIV-positive and HIV-negative women.

One of the strengths of this review is that it highlights diverse studies conducted in South Africa on breastfeeding in the era of HIV among both HIV-infected and uninfected women, and that EBF rates are subpar in both groups as a result of the premature introduction of liquids and solids. However, there is a dearth of policy-responsive implementation research to inform how the more recent HIV infant feeding national guidelines can be successfully implemented in South Africa, a country where this should be possible because there is now almost universal access to ART among HIV-positive women [93–96], and a call for strong protection, promotion and support for breastfeeding including those who are HIV-positive [33, 97]. As it has been found in other countries [98–101], our findings suggest that strong Code enforcement combined with increased investments in breastfeeding protection, promotion and support programs in South Africa are needed for the country to make progress towards meeting the United Nations target to increase breastfeeding rates to at least 50% by 2025 [17].

Because the infant formula distribution program was coordinated by the health sector, the findings from our review are highly consistent with previous studies showing that health workers play an important and influential role in counseling or advising mothers on infant feeding practices [77, 102–106].

Therefore, it is key that the WHO Code is properly enforced to allow for the environment conditions surrounding mothers in South Africa to become stronger enablers of optimal breastfeeding practices [106]. Unfortunately, in our review we did not find evidence that this has already started to happen yet in South Africa.

Although penalties exist for first and subsequent violations of regulation R991, with the first penalty being a fine and at most 6 months imprisonment [107], to date no individual or organization has been prosecuted for Code violations, a strong indicator of lack of enforcement or commitment to change the status quo. Thus, routine monitoring of this key legislation is required as encouraged by other academics and advocates [33, 97, 108]. As such, enforcing regulation R991, particularly the outreach to health workers by BMS manufacturers and their associates is imperative.

## Conclusion

The free distribution of infant formula combined with the BMS industry’s marketing practices that violate the WHO Code have played a role in suboptimal breastfeeding practices among both HIV-positive and negative women in South Africa [33, 109, 110].

This scoping review integrated evidence on infant feeding practices, especially EBF rates among HIV-positive and HIV-negative women in South Africa in the context of rapidly evolving HIV infant feeding guidelines from the WHO. Although highly effective ARTs has made breastfeeding for HIV-positive women safe, and South Africa has widespread access to ART [93, 95, 96], it is discouraging that women continue to cite fear of HIV transmission to their infants as a reason for either not breastfeeding or doing so for short periods of time. This finding calls for improved access to high quality breastfeeding counselling, education and awareness campaigns from the local health care facility to the national level.

Although monitoring and enforcement of the Code remain nonexistent, there has been progress in strengthening legislation. The most recent Status Report (2020) from WHO, UNICEF and IBFAN found 70% (136/194) of WHO Member States had new Code legislation [111]. However, only 31 countries regulated milk products for infants up to at least 36 months. Moreover, just 58% (79/136) ban the promotion of BMS at health facilities [111].

Even though enforcing the most recent WHO guidelines and the WHO Code are necessary to improve breastfeeding outcomes in South Africa, they are not sufficient because as our review shows, there are additional barriers that impact breastfeeding outcomes, including lack of social support among women returning to work or school after the birth of their children.

Mixed-methods research, including in-depth interviews with key informants representing different government sectors and civil society is needed to prioritize actions and strategies to make this happen in South Africa. This effort should be followed by implementation research and policy instruments [112, 113] that can guide South Africa in its efforts to scale up the protection, promotion, and support of breastfeeding programs at the national level in the context of the HIV pandemic.

## Data Availability

Data sharing not applicable to this article as no datasets were generated or analyzed during the current study.
